# The Influence of Chronic Pain and Cognitive Function on Spatial-Numerical Processing

**DOI:** 10.3389/fnbeh.2018.00165

**Published:** 2018-08-03

**Authors:** Melanie Spindler, Katharina Koch, Elena Borisov, Jale Özyurt, Peter Sörös, Christiane Thiel, Carsten Bantel

**Affiliations:** ^1^Department of Anesthesiology, Critical Care, Emergency Medicine and Pain Management, Medicine and Health Sciences, Carl von Ossietzky Universität Oldenburg, Oldenburg, Germany; ^2^Department of Psychology, Biological Psychology Lab, School of Medicine and Health Sciences, University of Oldenburg, Oldenburg, Germany; ^3^Wrocław Medical University, Wrocław, Poland; ^4^Department of Neurology, School of Medicine and Health Sciences, Carl von Ossietzky Universität Oldenburg, Oldenburg, Germany

**Keywords:** chronic pain, number sense, pain rating scales, number line task, pain assessment

## Abstract

Chronic pain (CP) is linked to changes in cognitive function. However, little is known about its influence on number sense, despite the fact that intact numerical-spatial processing is a prerequisite for valid scale-based pain assessments. This study aimed to elucidate whether number sense is changed in CP, to determine if changes have an impact on pain assessments using pain rating scales and what patient factors might contribute. *N* = 42 CP patients and *n* = 42 matched controls were analyzed (age range: 33–68 years). Numerical-spatial abilities were investigated by using number line tasks, where participants either estimated the position of a given number (position marking) or the value of a predefined mark (number naming). Pain intensity was assessed using numerical rating (NRS), verbal rating (VRS), and visual analog (VAS) scales. Additional measures included attention and working memory, verbal intelligence, medication and depression. Results revealed that in number naming, patients deviated more from expected (correct) responses than controls, and that VAS scores were significantly higher than both NRS and VRS and correlated with deviations in position making. Changes in number naming were predicted by pain intensity, sex and IQ but not by attention, memory or opioid medication. This article presents new insight on which cognitive mechanisms are influenced by CP with the focus on numerical spatial abilities. It could therefore provide useful knowledge in developing new pain assessment tools specifically for patients suffering from CP.

## Introduction

A mutual relationship between chronic pain (CP) perception and cognitive impairments, e.g., in attentional mechanisms, memory or executive functioning has repeatedly been reported (Wiech et al., 2008; Moriarty et al., 2011). Tamburin et al. (2014) for instance described altered decision making in a gambling task in patients with chronic back pain, while Oosterman et al. (2012) found sustained attention deficits in a group of patients with different CP conditions. Further, a recent systematic review confirmed a high likelihood for CP patients to experience moderate memory impairments (Kolb et al., 2012; Berryman et al., 2013) described “neglect-like" symptoms in patients with complex regional pain syndrome.

Still, another, albeit under-recognized but potentially clinically important cognitive modality, is the number sense. Number sense refers to the intuitive skill to estimate and compare numerical magnitudes and orders and is thought to be based on the “mental number line,” a spatial representation of numbers in the human brain. As it allows people to manipulate numbers as well as non-numerical quantities in form of rounding and approximations as well as exact calculations and measurements, it is suggested to be a key basic skill in today’s societies (Dehaene, 2011). In the clinical context, number sense is essential when scales such as the visual analog scale (VAS) or numerical rating scale (NRS) are used to assess pain intensity. In order to produce accurate results in those tasks, numerical-spatial abilities need to be intact (Lichtner et al., 2015). Wolrich et al. (2014), our group provided first evidence that this might not be the case in chronic compared to acute pain patients, as they used higher numbers to describe pain intensity in moderate and severe pain. Further, using left-to-right oriented number lines, we showed that patients compared to healthy controls deviated significantly more from the expected numerical value when asked to mark the respective spatial representation of a presented number on a line. However, this study raised several additional questions which we aimed to answer in the current study. For instance, as we only tested number sense with traditional left-to-right number lines, training effects could have occurred, resulting in decreased task difficulty over time. To counteract against training and habituation and to keep the task difficulty constant for the whole duration of the task, we added non-conventional (e.g., right-to-left, top-to-bottom) number lines. Further, as optical clues (landmarks) are used every day to help with the assessment of distances and proportions (Siegler and Opfer, 2003) the influence of these landmarks on results of number sense tests requires additional evaluation. It also remains elusive how other variables, which are traditionally linked to cognitive function that might be altered in CP conditions (such as attention, intelligence, or working memory), could influence performance of CP patients in number sense experiments. Finally, the impact of an altered number sense on the accuracy of responses to clinically used pain assessment tools needs to be evaluated.

Hence, the aims of the present study were (a) to assess whether number sense is changed in CP patients compared to healthy controls; (b) to assess the impact of possible number sense changes on the accuracy of responses of pain assessment tools; and (c) to evaluate whether changes in number sense are associated with performance in attention, intelligence or memory tests.

## Materials and Methods

### Participants

Chronic pain (CP) patients were recruited from the pain clinic of the Klinikum Oldenburg AöR, Germany, between March and August 2016. In addition, healthy controls and further patients were recruited through advertisement in a local newspaper. To ensure that results were not biased by specific demographic characteristics of participants, we matched pain patients and healthy controls for sex and age (±3 years). Inclusion criteria for patients were CP, perceived for at least 12 months (CP group), and for controls the absence of any pain related issues (healthy controls; C). Because the direction of the mental number line is presumed to depend on cultural background, resulting in a left-to-right oriented representation for persons from cultures with left-to-right reading and writing, and from right-to-left in cultures with right-to-left reading, respectively (Pitt and Casasanto, 2014), we only included participants who were born and grown up in Germany. Exclusion criteria for patients and controls were the following health conditions: impaired kidney function, chronic liver diseases, cancer, neurological diseases (such as dementia, epilepsy, migraine, Parkinson’s disease, multiple sclerosis, concussion, previous stroke), psychiatric disorders (such as schizophrenia or major depression) or alcohol- or drug-abuse. All participants had normal or corrected-to-normal vision and received 10€/h for their participation. This study was carried out in accordance the recommendations of the ICH-Good Clinical Practice Guideline (1996). The study was approved by the ethics committee of Carl von Ossietzky University of Oldenburg, Germany (Drs. 25/2015). All participants gave written informed consent prior to participating in the study. Testing was conducted in the afternoon between two and five pm in a standardized setting adjacent to the pain clinic at Klinikum Oldenburg.

### Experimental Tests and Questionnaires

#### Number Sense Experiments

##### Position marking

**FIGURE 1:**
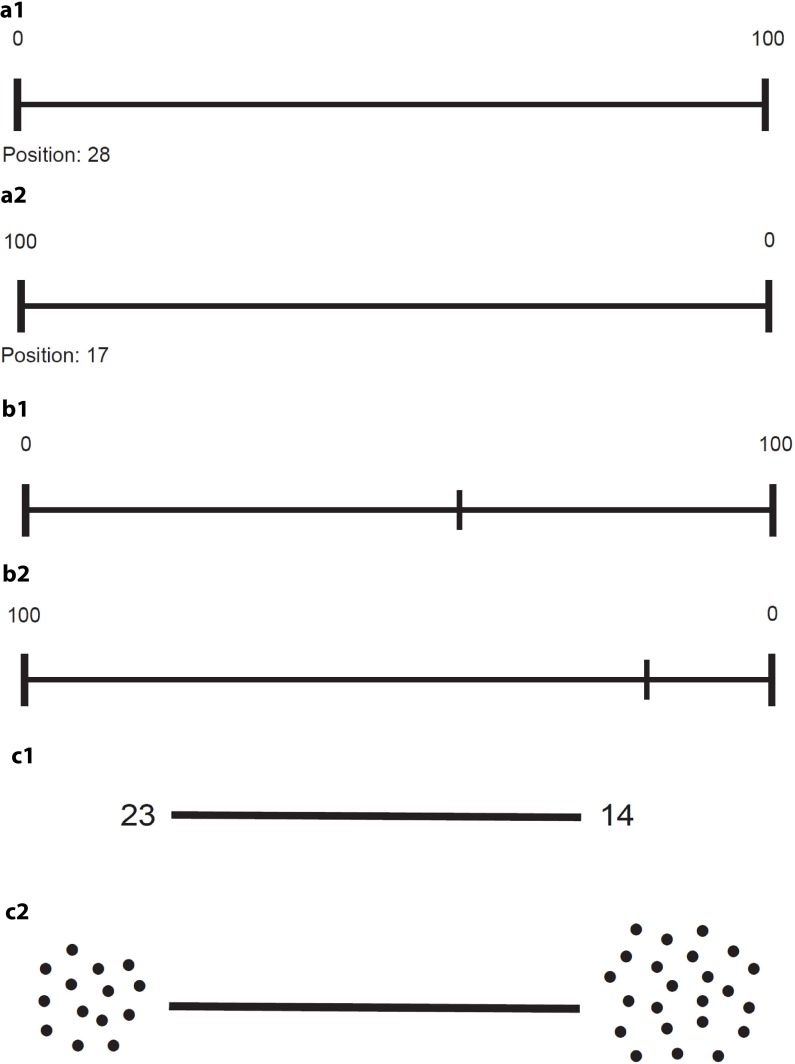
Examples for the number sense tasks position marking (a1,a2), number naming (b1,b2) and line bisection (c1,c2). a1 and b1 show a number line arranged in a ’familiar’ left to right fashion; a2 and b2 show a line in an ’unfamiliar’ right to left way. c1 displays quantities using numeric symbols whereas c2 uses non-symbolic dot-arrays.

For this task, each participant was exposed to a total of 16 23 cm long number lines printed on separate A4 papers. The ends of each line were anchored with “0” and “100,” respectively. Lines were presented horizontally (*n* = 8) and vertically (*n* = 8) with the anchors shown in a “familiar” or “unfamiliar” order. In “familiar” order “0” was either shown on the left of horizontal or at the bottom of vertical number lines. This was reversed in “unfamiliar” conditions where “0” was shown on the right (horizontal lines) or at the top (vertical lines), respectively. Lines were presented in random order and participants were asked to mark each line at the position which they thought would best represent a given number (6, 12, 17, 24, 28, 33, 37, 42, 58, 63, 67, 72, 76, 83, 88, 94) (**Figure 1**).

##### Number naming

Similar to position marking, each participant was exposed to a total of 16 23 cm long number lines printed on separate sheets of A4 paper. The lines were again presented either horizontally or vertically and anchored with “0” and “100” at each end in familiar and unfamiliar order, respectively. In contrast to position marking, the lines in this experiment were pre-marked and participants needed to indicate the numbers they thought these marks represented (**Figure 1**). To evaluate the influence of landmarks on the results obtained in the number naming and position marking tasks, items were ordered according to their distance to the nearest clue (line end anchored with 0 or 100, respectively, line mid-point). Thus, eight numbers were categorized as “low distance” (within a 13-unit distance from the landmark), and 8 as “high distance” (between 17 and 24 units from a landmark).

Both tests were used to assess differences in numerical-spatial processing between pain patients and healthy controls, and were conducted as paper- and pencil tests. The outcome measure was the mean absolute deviation from their expected responses (MADER) as described before (Wolrich et al., 2014). In short, responses were subtracted from their corresponding expected values.

##### Line bisection

This task served as a control task to rule out possible influences of spatial neglect on the number line tasks (Doricchi et al., 2005). Each participant was shown four 8 cm long horizontal lines in random order presented on separate A4 papers. Lines were either anchored with the Arabic digits “14” and “23” at each end (item A and B) or with two arrays of 14 and 23 dots (item C and D). The anchors were chosen to provide a numerical interval of 9, which according to Zorzi et al. (2002) was the distance with the highest sensitivity to show deficits in patients with neglect. In two items (A, C), the smaller quantity was presented on the left while in the remaining items (B, D) it was shown on the right (**Figure 1**). Participants were instructed to indicate the middle of each line as accurately as possible. The outcome measure was the mean deviation from the midline in mm.

#### Cognitive Testing

##### Attention and memory

Participants completed three subtests of the computerized TAP battery 2.3 (Zimmermann and Fimm, 2012). All tests were administered on a Dell Latitude E5550 (15.6″, 60 Hz, 1,366 × 768).

##### Sustained attention

This task measures control of an attentional focus and concentration over a longer time period (15 min). Participants were shown stimuli comprising sequences of symbols in different color, size, and filling. If a stimulus matched the preceding stimulus in either color or shape, the stimulus was considered a target stimulus and the participant was instructed to press a button as quickly as possible. An increase in the number of omissions over time indicates a decline in the ability to concentrate or focus on the task. Outcome measures were the number of omissions across the whole duration of task performance (15 min) and across each of three consecutive time bins with a 5 min duration (0–5, 5–10, and 10–15 min).

##### Covert shift of attention

This task assesses the ability to shift the focus of visual attention onto the peripheral space without changing the direction of the gaze. Participants were briefly presented a cue (arrow) which pointed either to the left or right-hand side of the screen until it disappeared. This cue was intended to direct the participant’s attention. Then, a target cross was shown either on the side which was indicated by the cue (valid cue, 80% of presentations), or on the opposite side (invalid cue, 20% of presentations). Participants were instructed to respond to the target stimulus by pressing a key as fast as possible, regardless of the validity of the cue. Outcome measures were mean reaction times for valid and invalid cues and for stimuli presented on the left or right side of the fixation point.

##### Working memory

This task probes the ability to control continuous updating of information flow in working memory. Participants were shown a sequence of 100 one-digit numbers at a constant rate. They were instructed to press a key as soon as possible whenever a stimulus matched the one that was presented two trials before (critical stimulus, 2-back). During the task, 15 critical stimuli were presented. The number of omissions and errors were recorded as outcome measures. Higher omission rates indicate a lack of control over the flow of information whereas an increased number of errors suggest attentional lapses.

##### Verbal intelligence

We used the Wortschatztest which is a standardized German vocabulary test with good correlation to general intelligence and education. In a multiple choice format, participants are required to identify, in each of 42 rows, a word within five non-words (Schmidt and Metzler, 1992). The sum of correctly identified words (highest score: 42) is transformed into standardized IQ scores according to the manual.

#### Assessment of Pain and Depression

##### Pain assessment

Chronic pain (CP) patients were asked to assess the average pain intensity which they had perceived during the last 24 h by using the following instruments: the VAS, which is presented as a 10 cm high pyramid with the top indicating “no pain” and the bottom “worst pain imaginable,” an 11-point NRS, with 0 representing “no pain” and 10 “worst pain imaginable” (Breivik et al., 2008), and a six-point verbal rating scale (VRS), that uses “no pain,” “slight pain,” “mild pain,” “moderate pain,” “severe pain,” and “worst pain imaginable” as verbal descriptors of pain intensity (Brunelli et al., 2010). For analysis the six-point VRS was converted to fit the common 0–10 scaling of the VAS and NRS scales as described elsewhere (Breivik et al., 2008; Brunelli et al., 2010). In short, the verbal descriptors were transformed into numerical values (no pain = 0; mild = 1; slight = 2; moderate = 3; severe = 4, worst pain imaginable = 5) and multiplied by 2. Transformed results were then used for comparison with results obtained with VAS and NRS, respectively.

##### Depression rating

We used the *Allgemeine Depressionsskala (ADS-K)* which is the short form of the German version of the Center for Epidemiological Studies Depression Scale (CES-D) (Radloff, 1977). The questionnaire consists of 15 items assessing depressive symptoms during the preceding week. Each item is answered on a four-point Likert scale with the following options: never or rarely (<1 day), sometimes (1–2 days), often (3–4 days), and always (5–7 days). The total score ranges from 0 to 45, and a score of >17 is considered clinically relevant (Hautzinger et al., 2012).

### Statistical Analysis

All participants with missing values were removed from the respective analyses: one participant was excluded from analysis of position marking, clinical pain assessment and covert shift attention. Also due to missing data, two patients were excluded from analysis of working memory and three from analysis of sustained attention. One additional participant was excluded from all number sense analyses, because of presenting as an extreme outlier (>7 standard deviations away from the overall mean). All analyses were performed with SPSS 24 (IBM, Ehningen, Germany). To detect differences between groups in cognitive function, number sense and intelligence relating to our first research question either independent samples *t*-tests or repeated measures analyses of variance (ANOVA) were used where appropriate. Within-group analyses were performed within the CP sample using dependent samples *t*-tests to investigate differences in results of pain rating scales. For correlation analyses we used Pearson correlation to explore the relationship of number sense tasks with pain rating scales.

Two stepwise linear regressions with forward selection were used to identify the relevance of demographical and cognitive factors for number sense performance as a dependent variable. We used the MADER for number naming overall as outcome variable based on the observed influence of landmarks in the position marking task. Participant demographics (age, sex, verbal IQ, group), as well as depression (ADS-K raw score), attentional measures (misses in the sustained attention task, validity of cue and position of target in the covert shift of attention) and working memory (misses) were included as predictors into the model. A second regression analysis used pain intensity and duration, and main pain syndrome (**Table 1**) to predict number sense performance within the CP sample.

**Table 1:** Characteristics of participants.

Characteristics	Controls	Chronic pain patients
Sample size; *n*	42	42
Gender (female); *n* (%)	31 (74)	31 (74)
Age [years]; mean (range)	54.1 (35–66)	54.0 (33–68)
Mean education^∗^ (*SD*)	2.71 (1.0)	2.05 (1.1)
Verbal IQ (*SD*)	106.0 (9.5)	98.0 (9.3)
Sleeping problems	8	28
Duration of pain [years]; mean (range)	/	16.8 (1–50)
Pain intensity^∗∗^ (SD)	/	5.9 (1.6)
Participants on opioid medication	/	15
Participants with depression (ADS-K score > 17)	1	19
Handedness (right, left, retrained left-handed)	39, 1, 2	37, 1, 4

**(Main) pain syndromes^†^**	**Controls**	**Chronic pain**

Fibromyalgia	/	9 (7)
Musculoskeletal back pain	/	20 (19)
Cervical/cervicobrachial pain	/	7 (5)
Neuropathic pain	/	3 (3)
Arthralgia	/	9 (6)
Abdominal pain	/	2 (2)
Myalgia	/	1 (0)

*SD, standard deviation; ADS-K, General Depression Scale – Short form; ^∗^education refers to 0 = no degree, 1 = lower secondary education, 2 = secondary school, 3 = A-levels, 4 = university degree; ^∗∗^on an 11-point Numerical Rating Scale (0 = no pain; 10 = worst pain imaginable) on the day of testing. ^†^The total amount of participants reporting different pain syndromes. In brackets, only the corresponding main pain category of each participant is listed.*

To correct for all measures of the number line tasks, which were the primary measures of the experiment, we used Bonferroni corrected alpha-levels of 0.005, two-sided (0.05/10). These comprised the overall performance, as well as horizontal and vertical lines, and lines in familiar and unfamiliar order of the numerical anchors. Additionally, Cohen’s *d* effect sizes were determined for each condition with *d* = 0.2 representing small effects, *d* = 0.5 medium effects, and *d* = 0.8 large effects (Cohen, 1988). For all other tests, a *p* < 0.05 (two-sided) was considered significant.

## Results

A total of *n* = 93 participants (50 controls and 43 patients) were invited to participate, but nine participants had to be excluded due to the following conditions: neurological or psychiatric diseases (*n* = 3), technical issues (*n* = 1), the absence of pain for more than 1 month (*n* = 1), lack of matching pain patients (*n* = 4). Thus, *n* = 84 (*n* = 42 healthy controls; *n* = 42 CP patients) could be included in our final analyses. Demographic and clinical variables of these patients and controls are depicted in **Table 1**. Compared to controls (C), chronic pain patients (CP) were similar with respect to age and sex. However, verbal IQ [*t*_(82)_ = 3.990, *p* < 0.001] and educational level [*t*_(80.884)_ = 2.817, *p* = 0.006] were significantly lower in CP patients and patients experienced significantly more depressive symptoms than controls [*M*_C_ = 5.2, *SD*_C_ = 3.8; *M*_CP_ = 16.1, *SD*_CP_ = 9.2; *t*_(54.519)_ = −7.111, *p* < 0.001].

### Experimental Tests and Questionnaires

#### Number Sense Experiments

Between-group analyses revealed that pain patients consistently had a significantly higher MADER in the overall condition (i.e., responses pooled across all conditions) and the vertically unfamiliar condition of the number naming task, with large effect sizes. Although no significant differences were observed in the overall position marking performance between pain patients and healthy controls, similar trends were noted here as well, with small to median effect sizes, and a significant difference in the horizontal unfamiliar condition (**Table 2**).

**Table 2:** Comparisons of MADER for different experimental conditions using independent samples *t*-tests.

Tasks	MADER (SD) controls	MADER (SD) patients	*T*-value	*df*	*p*-value	Cohen’s *d*
**Position Marking**						
Overall	4.1 (1.5)	5.1 (1.9)	-2.686	80	0.009	0.58
**Familiar**						
Horizontal	3.7 (1.7)	4.2 (2.1)	-1.217	81	0.227	0.26
Vertical	3.7 (1.8)	4.4 (2.0)	-1.852	81	0.068	0.37
**Unfamiliar**						
Horizontal	3.8 (2.0)	5.3 (2.4)	-3.288	81	0.001^∗^	0.60
Vertical	5.0 (2.6)	6.1 (2.9)	-1.782	80	0.079	0.40
**Number Naming**						
Overall	3.4 (0.9)	4.4 (1.4)	-4.075	68.205	<0.001^∗^	0.85
**Familiar**						
Horizontal	3.1 (1.3)	4.1 (2.4)	-2.298	81	0.024	0.52
Vertical	3.5 (1.4)	4.2 (1.6)	-1.987	81	0.05	0.47
**Unfamiliar**						
Horizontal	3.5 (1.3)	4.6 (2.1)	-2.813	81	0.006	0.63
Vertical	3.4 (1.2)	5.0 (1.9)	-4.392	67.147	<0.001^∗^	1.00

*On the left, the Mean Absolute Deviation from the Expected Respective Response (MADER) is shown for each subtask of number line experiments for controls and pain patients. On the right, results of statistical analyses for differences between group MADERs for each experimental condition are displayed. SD, standard deviation; ^∗^*p* < 0.005 (Bonferroni-corrected alpha-level).*

Evaluating the influence of landmarks on results of the number naming task, we found that there were no differences between high- and low-distance stimuli in both, controls and CP patients. In position marking, however, patients were significantly more accurate with low-distance than with high-distance stimuli. A similar but non-significant trend was observed for controls (**Table 3**).

**Table 3:** MADER and dependent *t*-statistics for low- and high-distance stimuli of the number line estimation tasks for chronic pain patients and controls.

	Number naming					Position marking				
MADER	Low distance	High distance	*T*	*df*	*p*	Low distance	High distance	*T*	*df*	*p*
MADER (SD) controls	3.5 (1.2)	3.3 (1.2)	–1.125	41	0.267	3.8 (1.7)	4.3 (1.7)	1.994	41	0.053
MADER (SD) patients	4.5 (1.6)	4.4 (1.9)	0.410	40	0.684	4.3 (1.7)	5.8 (2.5)	4.860	39	<0.001

##### Line bisection

We first determined whether responses differed depending on the type of stimulus (symbolic: Arabic digits vs. non-symbolic: dot arrays) participants were exposed to. Across all participants, response accuracy for stimulus type was not different for lines anchored with the smaller quantity on the right [symbolic item B vs. non-symbolic item D; *M*_B_ = 0.07, *SD*_B_ = 2.3; *M*_D_ = −0.06, *SD*_D_ = 2.03; *t*_(82)_ = 0.531, *p* = 0.597] and for those anchored with the smaller quantity on the left [symbolic item A vs. non-symbolic item C; *M*_A_ = 0.55, *SD*_A_ = 2.14; *M*_C_ = 0.60, *SD*_C_ = 2.18, *t*_(82)_ = −0.302, *p* = 0.764]. As the line anchor type did not seem to influence response accuracy, we pooled data obtained with the small symbolic and non-symbolic quantities on the left (MADER_left_), and those obtained with the smaller quantities on the right (MADER_right_). A 2 × 2-repeated-measures ANOVA was employed with *group* (chronic pain patients vs. controls) as between subjects and *MADER* (left vs. right) as within subject factor. Results revealed a non-significant main effect of *group* [*F*_(1)_ = 1.778, *p* = 0.186], a significant main effect of MADER [*F*_(1)_ = 15.626, *p* < 0.001], but no interaction between *group* and *MADER* [*F*_(1)_ = 0.178, *p* = 0.674; MADER_left_: *M*_CP_ = 0.88, *SD*_CP_ = 2.32; *M*_C_ = 0.29, *SD*_C_ = 1.68; MADER_right_: *M*_CP_ = 0.24, *SD*_CP_ = 1.91; *M*_C_ = −0.23, *SD*_C_ = 1.77], suggesting that visual-spatial attention was not different between groups.

##### Clinical pain assessment and number sense

**FIGURE 2:**
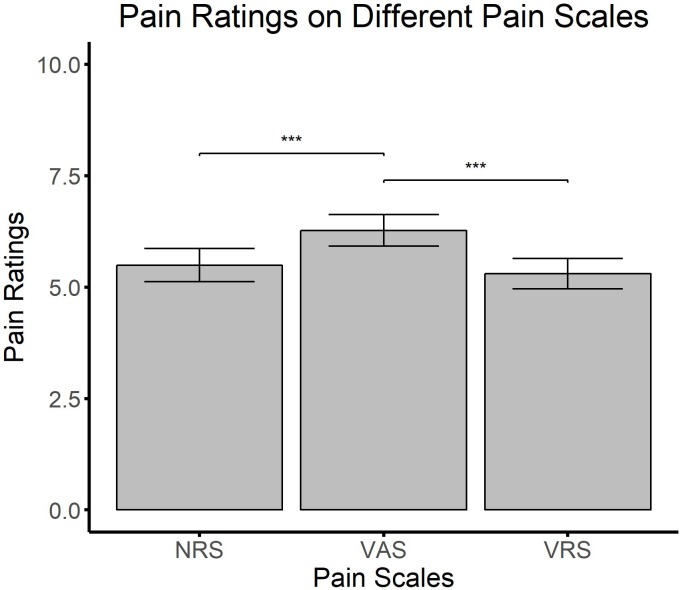
The mean and standard deviation for pain intensity statements for the three different scales. ^∗∗∗^*p* < 0.001.

To determine possible differences between pain rating scales, CP patients were included in a repeated measures ANOVA with *scale* (VAS, VRS, NRS) as within subject factor. Results showed a significant main effect of scale [*F*_(2,82)_ = 11.834, *p* < 0.001, $\eta_{\text{p}}^{2}$ = 0.224] (**Figure 2**). *Post hoc* dependent *t*-tests between scales revealed that VAS resulted in significantly higher intensity scores than both, NRS [*t*_(41)_ = −4.250, *p* < 0.001] and VRS [*t*_(41)_ = −4.326, *p* < 0.001]. However, there was no difference between the VRS and NRS [*t*_(41)_ = −0.374, *p* = 0.710].

To further examine the relationship between numerical tasks and VAS, correlation analyses including all CP patients were employed between overall number naming and VAS scores, and overall position marking and VAS scores. Results showed that only deviations in position marking (*r* = 0.321, *p* < 0.05), but not in number naming (*r* = 0.161, *p* = 0.308) were associated with higher values in pain ratings employing VAS.

Finally, the role of opioid medication on number sense performance was evaluated, suggesting that patients with opioid medication performed equally well on both number naming [*n* = 13; *M* = 4.7, *SD* = 1.7, *t*_(40)_ = −0.542, *p* = 0.591] and position marking [*n* = 13; *M* = 4.8, *SD* = 1.3, *t*_(39)_ = 0.818, *p* = 0.419] compared to patients without opioid medication (number naming: *n* = 29; *M* = 4.4, *SD* = 1.3; position marking: *n* = 28; *M* = 5.3, *SD* = 2.1).

### Cognitive Tests

#### Verbal IQ

To assess the influence of verbal IQ on participants’ performance on number sense tasks, the relationship of verbal IQ with the MADER of position marking and number naming was determined separately for pain patients and controls. In patients, both, overall number naming (*r* = −0.512, *p* < 0.01) and overall position marking (*r* = −0.359, *p* < 0.05) correlated negatively with IQ. However, in healthy controls (number naming: *r* = −0.179, *p* = 0.257; position marking: *r* = −0.227, *p* = 0.148) no such relationship could be found.

**Table 4:** Descriptive results from the subtests of the computerized TAP battery for chronic pain patients and controls separately.

Neuropsychological tests	Controls M (SD)	Chronic pain patients M (SD)
**Covert Shift of Attention**		
Valid trial–right target	316.0 (63.0)	323.0 (59.1)
Valid trial–left target	322.6 (67.1)	326.8 (67.8)
Invalid trial–right target	374.2 (88.0)	379.8 (75.8)
Invalid trial–left target	352.0 (91.8)	355.6 (72.0)
**Sustained Attention**		
Omissions 0–5 min	3.0 (2.9)	2.9 (2.5)
Omissions 5–10 min	3.0 (2.6)	4.0 (3.6)
Omissions 10–15 min	2.7 (2.5)	3.7 (3.3)
**Working Memory**		
Errors	1.7 (2.0)	2.7 (3.2)
Misses	1.3 (1.6)	1.7 (2.6)

*In covert shift of attention, values are given in milliseconds. For sustained attention and working memory, absolute values are reported.*

#### Subtests of the TAP Battery: Attention and Memory

##### Covert shift of attention

Results of the repeated measures ANOVA showed no significant difference between healthy controls and CP patients [main effect of *group*; *F*_(1)_ = 0.110, *p* = 0.741] in their time to detect left and right targets [interaction of *group* and *validity of cue*; *F*_(1,81)_ = 0.016, *p* = 0.899], as well as in their ability to shift their attention to both sides [interaction of *group* and *position of target*; *F*_(1,81)_ = 0.182, *p* = 0.671; **Table 4**].

##### Sustained attention

Results for the main effect of *group* [*F*_(1)_ = 1.168, *p* = 0.283] as well as for the interaction of *group* with *measurement-interval* [*F*_(1.842,145.554)_ = 3.009, *p* = 0.057] showed no significant differences between CP patients and healthy controls (**Table 4**).

Still, since there was a trend toward higher omissions of patients compared to controls over time, we employed a correlation analysis between the total number of omissions and overall number naming performance (MADER). Results suggest no significant relationship between number of omissions and number naming performance (*r* = 0.173, *p* = 0.116).

##### Working memory

There were no significant differences between healthy controls and CP patients in the number of errors [*t*_(65.572)_ = −1.591, *p* = 0.117] and misses [*t*_(80)_ = −0.764, *p* = 0.447] for this task (**Table 4**).

### Forward Stepwise Regression Analyses

Due to the finding that number naming performance was uninfluenced by landmarks compared to position marking experiments, only the overall MADER of the number naming tasks was used as a dependant variable.

**Table 5:** Overall number naming MADER regressed on different participant and pain characteristics using two stepwise regression analyses.

Predictor	β	*SE*	Standardized β	*t*	*p*
**Participant characteristics and cognition**					
IQ	−0.045	0.013	−0.352	−3.422	<0.01
Group	0.720	0.266	0.278	2.707	<0.01
Gender	0.663	0.282	0.222	2.353	<0.05
Constant	7.712	1.418		5.440	<0.001
**Pain-related variables**					
Pain intensity	0.356	0.136	0.401	2.624	<0.05
Constant	2.402	0.824		2.915	<0.01

*The upper panel shows results of the regression within both groups. Overall model fit: *R*^2^ = 0.325, Adjusted *R*^2^ = 0.298. The lower panel shows results of the regression analysis within the chronic pain sample. Overall model fit: *R*^2^ = 0.161, Adjusted *R*^2^ = 0.137.*

For the pain-unrelated regression, results showed that of all demographic and cognitive variables, only IQ, group and sex were included into the final model and in combination explained 29.8% (adjusted *R*^2^) variance of number naming performance [*F*_(3,76)_ = 12.172, *p* < 0.001] (**Table 5**).

For the pain-related regression within the CP sample, results showed that out of pain duration, pain syndrome and pain intensity, only pain intensity [*F*_(1,40)_ = 9.205, *p* < 0.01] significantly predicted number naming performance (adjusted *R*^2^ = 0.167) (**Table 5**).

## Discussion

The present study aimed to assess whether number sense is changed in CP patients compared to healthy controls. Using different variants of a number naming and position marking task, we found number naming to be more effective in detecting differences between C and CP participants than position marking. Here, patients deviated more from the expected value compared to healthy controls, with medium to large effect sizes. Although similar but not statistically significant trend was observed in overall position marking, we assume that patients were able to compensate and hence improve their accuracy in this task through the use of landmarks. Our results are well in line with findings of Wolrich et al. (2014), showing impaired numerical-spatial abilities in CP patients. In addition, based on findings indicating the successful use of landmarks as well as unimpaired line bisection performance in our patients, we conclude that mapping of the numerical information onto space is affected rather than spatial processing itself.

However, number sense assessments are new to the research on cognitive consequences of CP (Wolrich et al., 2014), and the use of number lines as part of a diagnostic process to uncover clinically relevant cognitive deficits would also be a novel approach, where further evaluation is needed to determine the most appropriate method for use in patients.

### Pain Intensity Assessments

Although often thought of as equivalent tools in assessing pain intensity (Breivik et al., 2008), results of our study, together with findings from Flaherty (1996) suggest that pain judgments obtained with VAS are higher than those of NRS and VRS. The reasons for these observations were unclear so far, but as VAS are similar to the position marking task of the present study, an altered number sense might contribute to the phenomenon. This is supported here by the positive correlation between VAS scores and the size of deviations in position marking. These results therefore suggest that number sense impairments in CP patients might be clinically relevant. Still, it remains unclear whether number sense impairments impact pain assessment using VAS in general, or whether increasing pain severity leads to increasing number sense changes. The NRS and VRS yielded similar responses, encouraging our assumption that not the numerical or spatial dimension of the task itself, but rather the interaction of both systems is impaired in patients. Whether this phenomenon is associated with other yet neglected factors (e.g., catastrophizing, arithmetic abilities) still needs to be determined. Our results suggest it would be advisable to assess pain intensity in CP not using only VAS, but to administer also other scales that do not rely on spatial-numerical processing, or to include landmarks on the VAS to aid with correct positioning. Newly developed scales should therefore also be tested using both an acute and a CP sample.

### Number Processing

There are two major components involved in the processing of numbers: each number can be perceived either in cardinality (its magnitude) or ordinality (its rank or position in a given sequence) (for a review of both systems see Lyons et al., 2016). Based on our results, we hypothesize that knowledge about cardinality (e.g., needed to give an accurate NRS score) is intact, but that patients are impaired in judging about ordinality (e.g., number naming and position marking, VAS). Number sense research suggests that in early childhood, cardinality is more important and better predicts arithmetic performance than ordinality, but that with ongoing age, ordinality becomes more relevant (Lyons et al., 2014). In view of this, a deficit in ordinality processing could be related to arithmetic achievement (Goffin and Ansari, 2016). Still, further research is needed to examine the origin and impact of the observed effect and its relation to other cognitive modalities.

### Co-variables for Number Sense

Interestingly, we found that age, depression, pain syndrome, and sleeping problems, as well as differences in attentional performance and working memory did not influence results in number sense tasks. Instead, we found group, pain intensity, verbal IQ and sex to be predictors for the number naming task. Especially the relationship between number sense impairment and lower IQ is worth mentioning as it might help to explain the origins of number sense changes in CP. Etherton and Tapscott (2015), for instance, hypothesized that cognitive impairment in CP patients might not be related to genuine pain mechanisms alone but also to comorbidities of CP, such as depression, sedative medications or sleep deprivation. Our results partially supported the notion of Etherton and Tapscott, because “pain syndrome” could not predict performance in number sense tasks. This suggests that other mechanisms independent of the original pain pathology are involved. However, as in our study attention and memory were not reduced in CP and as patients with opioid medications did not perform differently from patients without opioids, an influence of medications on our results was unlikely.

A possible link between IQ and CP was also supported by Spauwen et al. (2016) who found that lower verbal IQ was associated with the development of neuropathic pain in patients with diabetes. Our results are in agreement with this idea as they show a relationship between IQ and number line performance for pain patients, and in addition show no relationship in healthy controls. Nevertheless, further research is needed to investigate the role of intelligence in CP. Another possible explanation for the lower verbal IQ and concomitant number sense impairment would be cognitive pre-aging. This notion is supported by previous studies demonstrating slower information processing (Lee et al., 2010) as well as reduced attention, memory, and executive functions in CP compared to healthy controls of the same age (Dick and Rashiq, 2007; Solberg Nes et al., 2009). Further, studies also indicate that the mental number line increases in accuracy over an age-period of 11–30 years, and declines afterward (Halberda et al., 2012). Cognitive pre-aging in CP would therefore impair number sense through less accurate mental number representations. In the present study, we did not find any further indicators for cognitive pre-aging, as working memory was similar between the groups, and patients compared to controls solely showed a trend toward higher omissions over time in sustained attention. However, most studies which focused on CP, assessed working memory by using backward memory span tasks instead of the n-back task used here. Both tasks are known to be only weakly correlated (Conway et al., 2005). Moreover, in a study investigating whether n-back performance shows convergent validity with the digit span backward task, n-back performance was found to be more strongly correlated with a test of speeded information processing (Trail-Making Test A) than with a non-speeded test of working memory (digit span backward) (Miller et al., 2009). As we did not test whether CP patients in our study are impaired in working memory span performance, we cannot exclude that differences in number line task performance resulted from impaired working memory span (Kuhn and Holling, 2014; Corso, 2018). Still, both IQ and pain intensity were significant predictors for number naming performance and their contribution to altered number sense abilities in CP should be further investigated in future studies.

### Study Limitations

Factors such as physical disability, catastrophizing and polypharmacy that have been linked to cognitive performance but were not assessed here, should be included in future studies to determine their impact on number sense (Jorge et al., 2009; Pedro et al., 2016; Coppieters et al., 2017). Despite their limited value as predictors of responses to number sense tasks here, the many different CP syndromes included in our study may add to unexplained variance. However, our results also show that numerical-spatial changes are present across different painful conditions, hence adding considerably to the evolving field of research into the cognitive consequences of CP. Further, the proposed association between number sense alterations and pain intensity measurements could be criticized for not acknowledging enough the complex nature of clinical pain assessment (Shah et al., 2015). Although it is certainly true that a patient’s subjective pain rating is influenced by a variety of factors, number sense might still be an important contributor, a notion clearly supported here. Additionally, the influence of working memory span on number sense performance in CP patients still needs to be determined.

## Conclusion

This study adds further evidence to the recently proposed hypothesis that number sense is altered in CP patients. It also suggests that number naming might be more sensitive than position marking to assess these changes in patients. In addition, as number sense changes were positively correlated to pain intensity (VAS) ratings, our finding of an altered number naming performance in patients might bear some clinical relevance. Finally, despite attention and working memory not contributing to the phenomenon, number sense changes in CP might nevertheless be the result of complex processes involving pain intensity judgments, sex, intelligence and possibly the working memory span which was not measured in this study.

## Data Availability

The anonymized raw data supporting the conclusions of this manuscript will be made available by the authors, without undue reservation, to any qualified researcher.

## Author Contributions

CB and CT designed the study, applied for funding, and the ethical approval. Both were also involved in preparing the manuscript. MS recruited participants, analyzed the data, and prepared the manuscript. KK recruited participants and prepared the manuscript. EB recruited participants and prepared the manuscript. JÖ and PS analyzed the data and prepared the manuscript.

## Conflict of Interest Statement

The authors declare that the research was conducted in the absence of any commercial or financial relationships that could be construed as a potential conflict of interest.

## References

[B1] Berryman C. Stanton T. R. Jane Bowering K. Tabor A. McFarlane A. Lorimer Moseley G. (2013). Evidence for working memory deficits in chronic pain: a systematic review and meta-analysis. *Pain* 154 1181–1196. 10.1016/j.pain.2013.03.002 23707355

[B2] Breivik H. Borchgrevink P. C. Allen S. M. Rosseland L. A. Romundstad L. Hals E. K. (2008). Assessment of pain. *Br. J. Anaesth.* 101 17–24. 10.1093/bja/aen103 18487245

[B3] Brunelli C. Zecca E. Martini C. Campa T. Fagnoni E. Bagnasco M. (2010). Comparison of numerical and verbal rating scales to measure pain exacerbations in patients with chronic cancer pain. *Health Qual. Life Outcomes* 8:42. 10.1186/1477-7525-8-42 20412579 PMC2868814

[B4] Cohen J. (1988). *Statistical Power Analysis for the Behavioral Sciences*, 2 Edn. Abingdon: Routledge.

[B5] Conway A. R. A. Kane M. J. Bunting M. F. Hambrick D. Z. Wilhelm O. Engle R. W. (2005). Working memory span tasks: a methodological review and user’s guide. *Psychon. Bull. Rev.* 12 769–786. 10.3758/BF03196772 16523997

[B6] Coppieters I. De Pauw R. Kregel J. Malfliet A. Goubert D. Lenoir D. (2017). Differences between women with traumatic and idiopathic chronic neck pain and women without neck pain: interrelationships among disability, cognitive deficits, and central sensitization. *Phys. Ther.* 97 338–353. 10.2522/ptj.20160259 28403431

[B7] Corso L. V. (2018). Working memory, number sense, and arithmetical performance. *Psicol.Teor. Prát.* 20 155–167. 10.5935/1980-6906/psicologia.v20n1p155-167 25157301

[B8] Dehaene S. (2011). *The Number Sense - How the Mind Creates Mathematics.* New York, NY: Oxford University Press.

[B9] Dick B. D. Rashiq S. (2007). Disruption of attention and working memory traces in individuals with chronic pain. *Anesth. Analg.* 104 1223–1229. 10.1213/01.ane.0000263280.49786.f5 17456678

[B10] Doricchi F. Guariglia P. Gasparini M. Tomaiuolo F. (2005). Dissociation between physical and mental number line bisection in right hemisphere brain damage. *Nat. Neurosci.* 8 1663–1665. 10.1038/nn1563 16261135

[B11] Etherton J. L. Tapscott B. E. (2015). Performance on selected visual and auditory subtests of the wechsler memory scale-fourth edition during laboratory-induced pain. *J. Clin. Exp. Neuropsychol.* 37 243–252. 10.1080/13803395.2014.1002756 25655774

[B12] Flaherty S. A. (1996). Pain measurement tools for clinical practice and research. *AANA J.* 64 133–140. 9095685

[B13] Goffin C. Ansari D. (2016). Beyond magnitude: judging ordinality of symbolic number is unrelated to magnitude comparison and independently relates to individual differences in arithmetic. *Cognition* 150 68–76. 10.1016/j.cognition.2016.01.018 26851638

[B14] Halberda J. Ly R. Wilmer J. B. Naiman D. Q. Germine L. (2012). Number sense across the lifespan as revealed by a massive Internet-based sample. *Proc. Natl. Acad. Sci. U.S.A.* 109 11116–11120. 10.1073/pnas.1200196109 22733748 PMC3396479

[B15] Hautzinger M. Bailer M. Hofmeister D. Keller F. (2012). *Allgemeine Depressionsskala*, 2nd. Edn. Göttingen: Hogrefe.

[B16] Jorge L. L. Gerard C. Revel M. (2009). Evidences of memory dysfunction and maladaptive coping in chronic low back pain and rheumatoid arthritis patients: challenges for rehabilitation. *Eur. J. Phys. Rehabil. Med.* 45 469–477. 20032904

[B17] Kolb L. Lang C. Seifert F. Maihofner C. (2012). Cognitive correlates of ”neglect-like syndrome” in patients with complex regional pain syndrome. *Pain* 153 1063–1073. 10.1016/j.pain.2012.02.014 22424691

[B18] Kuhn J. T. Holling H. (2014). Number sense or working memory? The effect of two computer-based trainings on mathematical skills in elementary school. *Adv. Cogn. Psychol.* 10 59–67. 10.5709/acp-0157-2 25157301 PMC4116755

[B19] Lee D. M. Pendleton N. Tajar A. O’Neill T. W. O’Connor D. B. Bartfai G. (2010). Chronic widespread pain is associated with slower cognitive processing speed in middle-aged and older European men. *Pain* 151 30–36. 10.1016/j.pain.2010.04.024 20646831

[B20] Lichtner V. Dowding D. Closs S. J. (2015). The relative meaning of absolute numbers: the case of pain intensity scores as decision support systems for pain management of patients with dementia. *BMC Med. Inform. Decis. Mak.* 15:111. 10.1186/s12911-015-0233-8 26703244 PMC4690343

[B21] Lyons I. M. Price G. R. Vaessen A. Blomert L. Ansari D. (2014). Numerical predictors of arithmetic success in grades 1–6. *Dev. Sci.* 17 714–726. 10.1111/desc.12152 24581004

[B22] Lyons I. M. Vogel S. E. Ansari D. (2016). On the ordinality of numbers: a review of neural and behavioral studies. *Prog. Brain Res.* 227 187–221. 10.1016/bs.pbr.2016.04.010 27339013

[B23] Miller K. M. Price C. C. Okun M. S. Montijo H. Bowers D. (2009). Is the n-back task a valid neuropsychological measure for assessing working memory? *Arch. Clin. Neuropsychol.* 24 711–717. 10.1093/arclin/acp063 19767297 PMC2770861

[B24] Moriarty O. McGuire B. E. Finn D. P. (2011). The effect of pain on cognitive function: a review of clinical and preclinical research. *Prog. Neurobiol.* 93 385–404. 10.1016/j.pneurobio.2011.01.002 21216272

[B25] Oosterman J. Derksen L. C. van Wijck A. J. Kessels R. P. Veldhuijzen D. S. (2012). Executive and attentional functions in chronic pain: does performance decrease with increasing task load? *Pain Res. Manag.* 17 159–165. 10.1155/2012/962786 22606680 PMC3401086

[B26] Pedro M. C. Mercedes M. P. Ramon L. H. Borja M. R. (2016). Subjective memory complaints in elderly: relationship with health status, multimorbidity, medications, and use of services in a population-based study. *Int. Psychogeriatr.* 28 1903–1916. 10.1017/S104161021600106X 27468825

[B27] Pitt B. Casasanto D. (2014). “Experiential origins of the mental number line,” in *Paper Presented at the 36th Annual Conference of the Cognitive Science Society, Austin, TX.*

[B28] Radloff L. S. (1977). The CES-D Scale - A self-report depression scale for research in the general population. *Appl. Psychol. Meas.* 1 385–401. 10.1177/014662167700100306 26918431

[B29] Schmidt K. Metzler P. (1992). *Wortschatztest (WST).* Weinheim: Beltz Test.

[B30] Shah S. Ho A. C. Kuehler B. M. Childs S. R. Towlerton G. Goodall I. D. (2015). Different measures, different outcomes? Survey into the effectiveness of chronic pain clinics in a London tertiary referral center. *J. Pain Res.* 8 477–486. 10.2147/JPR.S80829 26346112 PMC4531003

[B31] Siegler R. S. Opfer J. E. (2003). The development of numerical estimation: evidence for multiple representations of numerical quantity. *Psychol. Sci.* 3 237–243. 10.1111/1467-9280.02438 12741747

[B32] Solberg Nes L. Roach A. R. Segerstrom S. C. (2009). Executive functions, self-regulation, and chronic pain: a review. *Ann. Behav. Med.* 37 173–183. 10.1007/s12160-009-9096-5 19357933

[B33] Spauwen P. J. Martens R. J. Stehouwer C. D. Verhey F. R. Schram M. T. Sep S. J. (2016). Lower verbal intelligence is associated with diabetic complications and slower walking speed in people with Type 2 diabetes: the Maastricht Study. *Diabet. Med.* 33 1632–1639. 10.1111/dme.13105 26926848

[B34] Tamburin S. Maier A. Schiff S. Lauriola M. F. Di Rosa E. Zanette G. (2014). Cognition and emotional decision-making in chronic low back pain: an ERPs study during Iowa gambling task. *Front. Psychol.* 5:1350. 10.3389/fpsyg.2014.01350 25505440 PMC4243494

[B35] Wiech K. Ploner M. Tracey I. (2008). Neurocognitive aspects of pain perception. *Trends Cogn. Sci.* 12 306–313. 10.1016/j.tics.2008.05.005 18606561

[B36] Wolrich J. Poots A. J. Kuehler B. M. Rice A. S. Rahman A. Bantel C. (2014). Is number sense impaired in chronic pain patients? *Br. J. Anaesth.* 113 1024–1031. 10.1093/bja/aeu255 25082664 PMC4235572

[B37] Zimmermann P. Fimm B. (2012). *Tests for Attentional Performance (TAP).* Herzogenrath: PsyTest.

[B38] Zorzi M. Priftis K. Umilta C. (2002). Brain damage: neglect disrupts the mental number line. *Nature* 417 138–139. 10.1038/417138a 12000950

