# Corrigendum: The Influence of Chronic Pain and Cognitive Function on Spatial-Numerical Processing

**DOI:** 10.3389/fnbeh.2019.00029

**Published:** 2019-02-19

**Authors:** Melanie Spindler, Katharina Koch, Elena Borisov, Jale Özyurt, Peter Sörös, Christiane Thiel, Carsten Bantel

**Affiliations:** ^1^Department of Anesthesiology, Critical Care, Emergency Medicine and Pain Management, Medicine and Health Sciences, Carl von Ossietzky Universität Oldenburg, Oldenburg, Germany; ^2^Department of Psychology, Biological Psychology Lab, School of Medicine and Health Sciences, University of Oldenburg, Oldenburg, Germany; ^3^Wrocław Medical University, Wrocław, Poland; ^4^Department of Neurology, School of Medicine and Health Sciences, Carl von Ossietzky Universität Oldenburg, Oldenburg, Germany

**Keywords:** chronic pain, number sense, pain rating scales, number line task, pain assessment

In the original article, there was a mistake in Tables 1–4 as published. The tables show the data for *n* = 37 chronic pain patients and *n* = 37 matched healthy controls. However, the tables should have shown data for *n* = 42 chronic pain patients and *n* = 42 matched healthy controls. The corrected Tables 1–4 appears below.

**Table 1:** Characteristics of participants.

**Characteristics**	**Controls**	**Chronic pain patients**
Sample size; *n*	42	42
Gender (female); *n* (%)	31 (74)	31 (74)
Age [years]; mean (range)	54.1 (35–66)	54.0 (33–68)
Mean education^*^ (SD)	2.71 (1.0)	2.05 (1.1)
Verbal IQ (SD)	106.0 (9.5)	98.0 (9.3)
Sleeping problems	8	28
Duration of pain [years]; mean (range)	/	16.8 (1–50)
Pain intensity^**^ (SD)	/	5.9 (1.6)
Participants on opioid medication	/	15
Participants with depression (ADS-K score >17)	1	19
Handedness (right, left, retrained left-handed)	39, 1, 2	37, 1, 4
**(Main) pain syndromes**^†^	**Controls**	**Chronic Pain**
Fibromyalgia	/	9 (7)
Musculoskeletal back pain	/	20 (19)
Cervical/cervicobrachial pain	/	7 (5)
Neuropathic pain	/	3 (3)
Arthralgia	/	9 (6)
Abdominal pain	/	2 (2)
Myalgia	/	1 (0)

SD, Standard deviation; ADS-K, General Depression Scale - Short form;

* education refers to 0 = no degree, 1 = lower secondary education, 2 = secondary school, 3 = A-levels, 4 = university degree;

** *on an 11-point Numerical Rating Scale (0 = no pain; 10 = worst pain imaginable) on the day of testing*.

† *The total amount of participants reporting different pain syndromes. In brackets, only the corresponding main pain category of each participant is listed*.

**Table 2:** Comparisons of MADER for different experimental conditions using independent samples t-tests.

**Tasks**	**MADER (SD) controls**	**MADER (SD) patients**	***T*-value**	***df***	***p*-value**	**Cohen's d**
**POSITION MARKING**						
Overall	4.1 (1.5)	5.1 (1.9)	−2.686	80	0.009	0.58
**Familiar**						
Horizontal	3.7 (1.7)	4.2 (2.1)	−1.217	81	0.227	0.26
Vertical	3.7 (1.8)	4.4 (2.0)	−1.852	81	0.068	0.37
**Unfamiliar**						
Horizontal	3.8 (2.0)	5.3 (2.4)	−3.288	81	0.001^*^	0.60
Vertical	5.0 (2.6)	6.1 (2.9)	−1.782	80	0.079	0.40
**NUMBER NAMING**						
Overall	3.4 (0.9)	4.4 (1.4)	−4.075	68.205	<0.001^*^	0.85
**Familiar**						
Horizontal	3.1 (1.3)	4.1 (2.4)	−2.298	81	0.024	0.52
Vertical	3.5 (1.4)	4.2 (1.6)	−1.987	81	0.05	0.47
**Unfamiliar**						
Horizontal	3.5 (1.3)	4.6 (2.1)	−2.813	81	0.006	0.63
Vertical	3.4 (1.2)	5.0 (1.9)	−4.392	67.147	<0.001^*^	1.00

On the left, the Mean Absolute Deviation from the Expected Respective Response (MADER) is shown for each subtask of number line experiments for controls and pain patients. On the right, results of statistical analyses for differences between group MADERs for each experimental condition are displayed. SD, standard deviation;

* *p < 0.005 (Bonferroni-corrected alpha-level)*.

**Table 3:** MADER and dependent t-statistics for low- and high-distance stimuli of the number line estimation tasks for chronic pain patients and controls.

**MADER**	**Number naming**					**Position marking**				
	**Low distance**	**High distance**	**T**	**df**	***p***	**Low distance**	**High distance**	**T**	**df**	***p***
MADER (SD) controls	3.5 (1.2)	3.3 (1.2)	−1.125	41	0.267	3.8 (1.7)	4.3 (1.7)	1.994	41	0.053
MADER (SD) patients	4.5 (1.6)	4.4 (1.9)	0.410	40	0.684	4.3 (1.7)	5.8 (2.5)	4.860	39	<0.001

**Table 4:** Descriptive results from the subtests of the computerized TAP battery for chronic pain patients and controls separately.

**Neuropsychological tests**	**Controls M (SD)**	**Chronic pain patients M (SD)**
**COVERT SHIFT OF ATTENTION**		
Valid trial–right target	316.0 (63.0)	323.0 (59.1)
Valid trial–left target	322.6 (67.1)	326.8 (67.8)
Invalid trial–right target	374.2 (88.0)	379.8 (75.8)
Invalid trial–left target	352.0 (91.8)	355.6 (72.0)
**SUSTAINED ATTENTION**		
Omissions 0–5 min.	3.0 (2.9)	2.9 (2.5)
Omissions 5–10 min.	3.0 (2.6)	4.0 (3.6)
Omissions 10–15 min.	2.7 (2.5)	3.7 (3.3)
**WORKING MEMORY**		
Errors	1.7 (2.0)	2.7 (3.2)
Misses	1.3 (1.6)	1.7 (2.6)

*In covert shift of attention, values are given in milliseconds. For sustained attention and working memory, absolute values are reported*.

Additionally, there was a mistake in the legend for Table 1 as published. The scaling of the variables “education” and “opioid medication” was incorrect. The correct legend appears below.

“SD: Standard deviation; ADS-K: General Depression Scale - Short form; ^*^education refers to 0 = no degree, 1 = lower secondary education, 2 = secondary school, 3 = A-levels, 4 = university degree; ^**^on an 11-point Numerical Rating Scale (0 = no pain; 10 = worst pain imaginable) on the day of testing.

†The total amount of participants reporting different pain syndromes. In brackets, only the corresponding main pain category of each participant is listed.”

Lastly, in the original article, there was an error. The number sense performance of patients with vs. without opioid medication, was compared using the same incorrect sample size as mentioned above.

A correction has been made to the ***Results***, ***Experimental Tests and Questionnaires***, ***Clinical pain assessment, and number sense***.

“Finally, the role of opioid medication on number sense performance was evaluated, suggesting that patients with opioid medication performed equally well on both number naming [*n* = 13; *M* = 4.7, *SD* = 1.7, *t*_(40)_ = −0.542, *p* = 0.591] and position marking [*n* = 13; *M* = 4.8, *SD* = 1.3, *t*_(39)_ = 0.818, *p* = 0.419] compared to patients without opioid medication (number naming: *n* = 29; *M* = 4.4, *SD* = 1.3; position marking: *n* = 28; *M* = 5.3, *SD* = 2.1).”

The authors apologize for these errors and state that they do not change the scientific conclusions of the article in any way. The original article has been updated.

## Conflict of Interest Statement

The authors declare that the research was conducted in the absence of any commercial or financial relationships that could be construed as a potential conflict of interest.

